# Poly[(μ_2_-4,4′-bipyridine-κ^2^ *N*:*N*′)bis­(μ_4_-cyclo­hexane-1,3-dicarboxyl­ato-κ^4^ *O*:*O*′:*O*′′:*O*′′′)dizinc(II)]

**DOI:** 10.1107/S1600536809035168

**Published:** 2009-09-09

**Authors:** Mohd. Razali Rizal, Seik Weng Ng

**Affiliations:** a Department of Chemistry, University of Malaya, 50603 Kuala Lumpur, Malaysia

## Abstract

The cyclo­hexane-1,3-dicarboxyl­ate dianion in the title three-dimensional coordination polymer, [Zn_2_(C_8_H_10_O_4_)_2_(C_10_H_8_N_2_)]_*n*_, has one carboxyl­ate group in an equatorial position and the other in an axial position of the cyclo­hexane ring, which adopts a chair conformation. The carboxyl­ate groups function as bridges to two adjacent Zn^II^ atoms, generating a layer motif. Adjacent layers are linked through the 4,4′-bipyridine *N*-heterocycle, forming a three-dimensional network; the geometry of Zn^II^ is square-pyramidal with the N atom of the *N*-heterocycle occupying the apical position. The *N*-heterocycle lies about a center of inversion and is disordered in a 1:1 ratio with respect to the C atoms bearing H atoms.

## Related literature

For the zinc cyclo­hexane-1,3-dicarboxyl­ate adduct of 1,10-phenanthroline, see: Bailey *et al.* (2008 ▶).
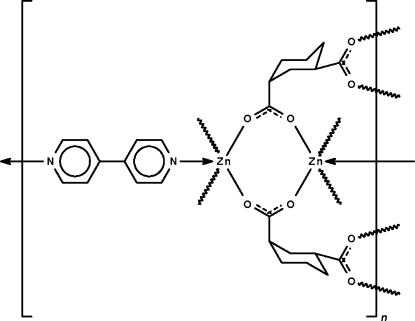


## Experimental

### 

#### Crystal data

[Zn_2_(C_8_H_10_O_4_)_2_(C_10_H_8_N_2_)]
*M* *_r_* = 313.64
Monoclinic, 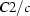

*a* = 22.251 (2) Å
*b* = 13.436 (1) Å
*c* = 8.552 (1) Å
β = 104.446 (5)°
*V* = 2475.8 (3) Å^3^
*Z* = 8
Mo *K*α radiation
μ = 1.99 mm^−1^
*T* = 100 K
0.12 × 0.02 × 0.02 mm

#### Data collection

Bruker SMART APEX diffractometer
Absorption correction: multi-scan (*SADABS*; Sheldrick, 1996 ▶) *T*_min_ = 0.557, *T*_max_ = 1.000
8536 measured reflections
2175 independent reflections
1407 reflections with *I* > 2σ(*I*)
*R*_int_ = 0.116

#### Refinement

*R*[*F* ^2^ > 2σ(*F* ^2^)] = 0.050
*wR*(*F* ^2^) = 0.123
*S* = 0.99
2175 reflections
184 parameters
36 restraints
H-atom parameters constrained
Δρ_max_ = 0.77 e Å^−3^
Δρ_min_ = −0.65 e Å^−3^

### 

Data collection: *APEX2* (Bruker, 2007 ▶); cell refinement: *SAINT* (Bruker, 2007 ▶); data reduction: *SAINT* program(s) used to solve structure: *SHELXS97* (Sheldrick, 2008 ▶); program(s) used to refine structure: *SHELXL97* (Sheldrick, 2008 ▶); molecular graphics: *XSHELL* (Sheldrick, 2008 ▶); software used to prepare material for publication: *publCIF* (Westrip, 2009 ▶).

## Supplementary Material

Crystal structure: contains datablocks global, I. DOI: 10.1107/S1600536809035168/tk2535sup1.cif

Structure factors: contains datablocks I. DOI: 10.1107/S1600536809035168/tk2535Isup2.hkl

Additional supplementary materials:  crystallographic information; 3D view; checkCIF report

## Figures and Tables

**Fig. 1.**
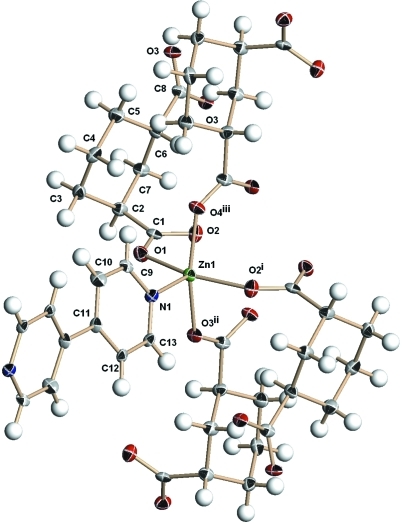
50% Probability displacement ellipsoid plot of the asymmetric unit of Zn2(C10H8N2)(C8H10O4)2 extended to show the coordination environment of the zinc center and a full molecule of 4,4'-bipyridine. Hydrogen atoms are drawn as spheres of arbitrary radius. Symmetry operations i: 0.5-x, 1.5-y, 1-z; ii: 0.5-x, 0.5+y, 0.5-z; iii: x, 1-y, 0.5+z.

## supplementary crystallographic information

### Experimental

Zinc acetate (0.15 g, 0.76 mmol), cyclohexane-1,3-dicarboxylic acid (mixture of *cis*- and *trans*-isomers) (0.13 g, 0.76 mmol) and 4,4'-bipyridine (0.12 g, 0.76 mmol) along with water (18 ml) were heated in a 23-ml Teflon-lined stainless-steel Parr bomb. The bomb was heated at 403 K for 3 days. The bomb was cooled to room temperature at 5 K per hour. Tiny crystals were isolated from the solution.

### Refinement

Hydrogen atoms were included in the refinement in the riding model approximation with C–H 0.95–1.00 Å, and with *U*(H) 1.2*U*_eq_(C). The 4,4'-bipyridine N-heterocycle is disordered about a center-of-inversion with respect to the carbon atoms bearing a hydrogen atom. As the disordered refined to nearly 50:50, the occupancy was fixed as 0.5. The distances of pairs of atoms were restrained to within 0.01 Å, and the pyridyl ring was restrained to near planarity. The displacement factors of the primed atoms were given those of the unprimed ones; the anisotropic behavior was restrained to be nearly isotropic.

### Crystal data

**Table:**

[Zn_2_(C_8_H_10_O_4_)_2_(C_10_H_8_N_2_)]	*F*(000) = 1288
*M**_r_* = 313.64	*D*_x_ = 1.683 Mg m^−^^3^
Monoclinic, *C*2/*c*	Mo *K*α radiation, λ = 0.71073 Å
Hall symbol: -C 2yc	Cell parameters from 543 reflections
*a* = 22.251 (2) Å	θ = 2.8–21.0°
*b* = 13.436 (1) Å	µ = 1.99 mm^−^^1^
*c* = 8.552 (1) Å	*T* = 100 K
β = 104.446 (5)°	Prism, colorless
*V* = 2475.8 (3) Å^3^	0.12 × 0.02 × 0.02 mm
*Z* = 8	

### Data collection

**Table:**

Bruker SMART APEX diffractometer	2175 independent reflections
Radiation source: fine-focus sealed tube	1407 reflections with *I* > 2σ(*I*)
graphite	*R*_int_ = 0.116
ω scans	θ_max_ = 25.0°, θ_min_ = 1.8°
Absorption correction: multi-scan (*SADABS*; Sheldrick, 1996)	*h* = −26→25
*T*_min_ = 0.557, *T*_max_ = 1.000	*k* = −15→15
8536 measured reflections	*l* = −10→10

### Refinement

**Table:**

Refinement on *F*^2^	Primary atom site location: structure-invariant direct methods
Least-squares matrix: full	Secondary atom site location: difference Fourier map
*R*[*F*^2^ > 2σ(*F*^2^)] = 0.050	Hydrogen site location: inferred from neighbouring sites
*wR*(*F*^2^) = 0.123	H-atom parameters constrained
*S* = 0.99	*w* = 1/[σ^2^(*F*_o_^2^) + (0.0478*P*)^2^] where *P* = (*F*_o_^2^ + 2*F*_c_^2^)/3
2175 reflections	(Δ/σ)_max_ = 0.001
184 parameters	Δρ_max_ = 0.77 e Å^−^^3^
36 restraints	Δρ_min_ = −0.65 e Å^−^^3^

### Fractional atomic coordinates and isotropic or equivalent isotropic displacement parameters (Å^2^)

**Table:**

	*x*	*y*	*z*	*U*_iso_*/*U*_eq_	Occ. (<1)
Zn1	0.30423 (3)	0.80190 (5)	0.58500 (8)	0.0148 (2)	
O1	0.33841 (19)	0.7868 (3)	0.3855 (5)	0.0226 (10)	
O2	0.25270 (18)	0.7072 (3)	0.2612 (4)	0.0220 (10)	
O3	0.24532 (18)	0.4228 (3)	0.0174 (4)	0.0192 (9)	
O4	0.33149 (18)	0.3418 (3)	0.1412 (5)	0.0196 (10)	
N1	0.3745 (2)	0.8727 (3)	0.7432 (5)	0.0157 (11)	
C1	0.3081 (3)	0.7349 (4)	0.2723 (7)	0.0182 (14)	
C2	0.3373 (3)	0.7034 (4)	0.1370 (7)	0.0190 (13)	
H2	0.3281	0.7563	0.0521	0.023*	
C3	0.4082 (3)	0.6950 (4)	0.1978 (7)	0.0207 (13)	
H3A	0.4265	0.6834	0.1052	0.025*	
H3B	0.4252	0.7581	0.2503	0.025*	
C4	0.4259 (3)	0.6096 (4)	0.3182 (7)	0.0218 (14)	
H4A	0.4716	0.6040	0.3530	0.026*	
H4B	0.4104	0.6239	0.4148	0.026*	
C5	0.3985 (3)	0.5113 (4)	0.2433 (7)	0.0232 (15)	
H5A	0.4182	0.4929	0.1556	0.028*	
H5B	0.4080	0.4584	0.3263	0.028*	
C6	0.3287 (3)	0.5179 (4)	0.1755 (7)	0.0183 (14)	
H6	0.3108	0.5325	0.2693	0.022*	
C7	0.3106 (3)	0.6056 (4)	0.0586 (7)	0.0194 (14)	
H7A	0.3261	0.5935	−0.0386	0.023*	
H7B	0.2648	0.6107	0.0240	0.023*	
C8	0.3000 (3)	0.4193 (4)	0.1038 (7)	0.0180 (14)	
C9	0.4299 (6)	0.8277 (15)	0.8020 (17)	0.015 (3)	0.50
H9	0.4346	0.7607	0.7715	0.019*	0.50
C10	0.4800 (11)	0.8744 (6)	0.9043 (19)	0.022 (3)	0.50
H10	0.5181	0.8400	0.9432	0.027*	0.50
C9'	0.4219 (6)	0.8212 (16)	0.8385 (15)	0.015 (3)	0.50
H9'	0.4213	0.7506	0.8346	0.019*	0.50
C10'	0.4713 (10)	0.8691 (6)	0.942 (2)	0.022 (3)	0.50
H10'	0.5040	0.8311	1.0076	0.027*	0.50
C11	0.4734 (2)	0.9726 (4)	0.9490 (6)	0.0155 (13)	
C12	0.4164 (6)	1.0188 (15)	0.8894 (17)	0.016 (3)	0.50
H12	0.4102	1.0856	0.9184	0.019*	0.50
C13	0.3687 (11)	0.9669 (6)	0.7878 (19)	0.014 (2)	0.50
H13	0.3301	0.9996	0.7477	0.017*	0.50
C12'	0.4238 (6)	1.0236 (16)	0.8504 (16)	0.016 (3)	0.50
H12'	0.4230	1.0943	0.8524	0.019*	0.50
C13'	0.3758 (10)	0.9721 (6)	0.750 (2)	0.014 (2)	0.50
H13'	0.3425	1.0085	0.6828	0.017*	0.50

### Atomic displacement parameters (Å^2^)

**Table:**

	*U*^11^	*U*^22^	*U*^33^	*U*^12^	*U*^13^	*U*^23^
Zn1	0.0119 (4)	0.0150 (4)	0.0150 (3)	−0.0020 (3)	−0.0011 (2)	−0.0008 (3)
O1	0.027 (2)	0.021 (2)	0.021 (2)	−0.0065 (19)	0.0087 (19)	−0.0056 (18)
O2	0.019 (2)	0.030 (3)	0.017 (2)	−0.002 (2)	0.0051 (18)	−0.0029 (18)
O3	0.011 (2)	0.018 (2)	0.023 (2)	0.0018 (18)	−0.0065 (19)	0.0021 (18)
O4	0.021 (3)	0.013 (2)	0.021 (2)	0.0026 (18)	−0.0026 (19)	−0.0014 (17)
N1	0.016 (3)	0.018 (3)	0.013 (2)	0.001 (2)	0.003 (2)	−0.003 (2)
C1	0.024 (4)	0.008 (3)	0.022 (3)	0.000 (2)	0.005 (3)	0.002 (2)
C2	0.023 (3)	0.017 (3)	0.016 (3)	0.000 (3)	0.004 (3)	−0.002 (3)
C3	0.017 (3)	0.020 (3)	0.025 (3)	−0.004 (3)	0.006 (3)	−0.005 (3)
C4	0.017 (4)	0.027 (4)	0.020 (3)	0.003 (3)	0.004 (3)	−0.005 (3)
C5	0.020 (4)	0.021 (3)	0.027 (4)	0.000 (3)	0.003 (3)	0.000 (3)
C6	0.015 (4)	0.017 (3)	0.021 (3)	−0.002 (3)	0.000 (3)	0.002 (3)
C7	0.024 (4)	0.019 (3)	0.014 (3)	−0.001 (3)	0.003 (3)	−0.003 (2)
C8	0.026 (4)	0.015 (3)	0.013 (3)	−0.001 (3)	0.005 (3)	0.001 (3)
C9	0.025 (5)	0.013 (4)	0.011 (6)	−0.001 (4)	0.008 (4)	0.005 (4)
C10	0.018 (6)	0.022 (4)	0.022 (7)	0.000 (3)	−0.004 (4)	0.007 (3)
C9'	0.025 (5)	0.013 (4)	0.011 (6)	−0.001 (4)	0.008 (4)	0.005 (4)
C10'	0.018 (6)	0.022 (4)	0.022 (7)	0.000 (3)	−0.004 (4)	0.007 (3)
C11	0.010 (3)	0.021 (3)	0.013 (3)	−0.002 (3)	−0.001 (3)	−0.001 (2)
C12	0.015 (4)	0.017 (3)	0.017 (6)	−0.002 (3)	0.009 (4)	−0.006 (4)
C13	0.008 (5)	0.018 (3)	0.015 (7)	0.001 (3)	0.000 (4)	−0.002 (3)
C12'	0.015 (4)	0.017 (3)	0.017 (6)	−0.002 (3)	0.009 (4)	−0.006 (4)
C13'	0.008 (5)	0.018 (3)	0.015 (7)	0.001 (3)	0.000 (4)	−0.002 (3)

### Geometric parameters (Å, °)

**Table:**

Zn1—O1	2.044 (4)	C4—H4B	0.9900
Zn1—O2^i^	2.045 (4)	C5—C6	1.519 (8)
Zn1—O3^ii^	2.034 (4)	C5—H5A	0.9900
Zn1—O4^iii^	2.044 (4)	C5—H5B	0.9900
Zn1—N1	2.031 (5)	C6—C8	1.532 (8)
Zn1—Zn1^i^	2.8517 (13)	C6—C7	1.533 (7)
O1—C1	1.246 (7)	C6—H6	1.0000
O2—C1	1.267 (7)	C7—H7A	0.9900
O2—Zn1^i^	2.045 (4)	C7—H7B	0.9900
O3—C8	1.257 (7)	C9—C10	1.384 (10)
O3—Zn1^iv^	2.034 (4)	C9—H9	0.9500
O4—C8	1.252 (7)	C10—C11	1.392 (10)
O4—Zn1^v^	2.044 (4)	C10—H10	0.9500
N1—C13'	1.338 (9)	C9'—C10'	1.385 (10)
N1—C13	1.338 (9)	C9'—H9'	0.9500
N1—C9'	1.351 (9)	C10'—C11	1.393 (10)
N1—C9	1.351 (9)	C10'—H10'	0.9500
C1—C2	1.522 (8)	C11—C12'	1.390 (10)
C2—C7	1.526 (7)	C11—C12	1.391 (10)
C2—C3	1.537 (8)	C11—C11^vi^	1.478 (11)
C2—H2	1.0000	C12—C13	1.380 (10)
C3—C4	1.526 (8)	C12—H12	0.9500
C3—H3A	0.9900	C13—H13	0.9500
C3—H3B	0.9900	C12'—C13'	1.380 (10)
C4—C5	1.526 (8)	C12'—H12'	0.9500
C4—H4A	0.9900	C13'—H13'	0.9500
			
N1—Zn1—O3^ii^	99.06 (17)	C6—C5—H5B	109.2
N1—Zn1—O2^i^	95.55 (16)	C4—C5—H5B	109.2
O3^ii^—Zn1—O2^i^	88.19 (16)	H5A—C5—H5B	107.9
N1—Zn1—O1	102.59 (16)	C5—C6—C8	112.8 (5)
O3^ii^—Zn1—O1	89.70 (15)	C5—C6—C7	111.8 (5)
O2^i^—Zn1—O1	161.85 (15)	C8—C6—C7	112.9 (5)
N1—Zn1—O4^iii^	98.71 (17)	C5—C6—H6	106.3
O3^ii^—Zn1—O4^iii^	162.21 (15)	C8—C6—H6	106.3
O2^i^—Zn1—O4^iii^	89.29 (16)	C7—C6—H6	106.3
O1—Zn1—O4^iii^	87.23 (15)	C2—C7—C6	111.4 (5)
N1—Zn1—Zn1^i^	169.00 (12)	C2—C7—H7A	109.3
O3^ii^—Zn1—Zn1^i^	82.66 (11)	C6—C7—H7A	109.3
O2^i^—Zn1—Zn1^i^	73.59 (11)	C2—C7—H7B	109.3
O1—Zn1—Zn1^i^	88.25 (12)	C6—C7—H7B	109.3
O4^iii^—Zn1—Zn1^i^	79.73 (11)	H7A—C7—H7B	108.0
C1—O1—Zn1	117.5 (4)	O4—C8—O3	125.4 (5)
C1—O2—Zn1^i^	135.7 (4)	O4—C8—C6	117.8 (5)
C8—O3—Zn1^iv^	124.3 (4)	O3—C8—C6	116.7 (5)
C8—O4—Zn1^v^	127.7 (4)	N1—C9—C10	123 (2)
C13'—N1—C9'	118.7 (15)	N1—C9—H9	118.4
C13—N1—C9'	115.4 (14)	C10—C9—H9	118.4
C13'—N1—C9	115.0 (15)	C9—C10—C11	119 (2)
C13—N1—C9	117.4 (15)	C9—C10—H10	120.6
C13'—N1—Zn1	120.0 (11)	C11—C10—H10	120.6
C13—N1—Zn1	121.4 (11)	N1—C9'—C10'	122 (2)
C9'—N1—Zn1	121.2 (11)	N1—C9'—H9'	119.2
C9—N1—Zn1	121.2 (11)	C10'—C9'—H9'	119.2
O1—C1—O2	123.4 (5)	C9'—C10'—C11	120 (2)
O1—C1—C2	119.6 (5)	C9'—C10'—H10'	119.8
O2—C1—C2	116.9 (5)	C11—C10'—H10'	119.8
C7—C2—C1	112.3 (5)	C12'—C11—C10	115.0 (14)
C7—C2—C3	109.8 (5)	C12—C11—C10	118.1 (14)
C1—C2—C3	111.1 (5)	C12'—C11—C10'	116.8 (14)
C7—C2—H2	107.8	C12—C11—C10'	114.3 (14)
C1—C2—H2	107.8	C12'—C11—C11^vi^	120.7 (12)
C3—C2—H2	107.8	C12—C11—C11^vi^	121.1 (12)
C4—C3—C2	110.7 (5)	C10—C11—C11^vi^	120.7 (12)
C4—C3—H3A	109.5	C10'—C11—C11^vi^	122.5 (11)
C2—C3—H3A	109.5	C13—C12—C11	120 (2)
C4—C3—H3B	109.5	C13—C12—H12	120.2
C2—C3—H3B	109.5	C11—C12—H12	120.2
H3A—C3—H3B	108.1	N1—C13—C12	123 (2)
C5—C4—C3	111.0 (5)	N1—C13—H13	118.5
C5—C4—H4A	109.4	C12—C13—H13	118.5
C3—C4—H4A	109.4	C13'—C12'—C11	120 (2)
C5—C4—H4B	109.4	C13'—C12'—H12'	119.8
C3—C4—H4B	109.4	C11—C12'—H12'	119.8
H4A—C4—H4B	108.0	N1—C13'—C12'	122 (2)
C6—C5—C4	111.9 (5)	N1—C13'—H13'	118.9
C6—C5—H5A	109.2	C12'—C13'—H13'	118.9
C4—C5—H5A	109.2		
			
N1—Zn1—O1—C1	171.8 (4)	C3—C2—C7—C6	56.6 (6)
O3^ii^—Zn1—O1—C1	−89.0 (4)	C5—C6—C7—C2	−54.5 (6)
O2^i^—Zn1—O1—C1	−5.7 (7)	C8—C6—C7—C2	177.0 (5)
O4^iii^—Zn1—O1—C1	73.5 (4)	Zn1^v^—O4—C8—O3	−3.1 (8)
Zn1^i^—Zn1—O1—C1	−6.3 (4)	Zn1^v^—O4—C8—C6	173.7 (3)
O3^ii^—Zn1—N1—C13'	−0.6 (6)	Zn1^iv^—O3—C8—O4	−0.9 (8)
O2^i^—Zn1—N1—C13'	−89.7 (6)	Zn1^iv^—O3—C8—C6	−177.8 (3)
O1—Zn1—N1—C13'	91.1 (6)	C5—C6—C8—O4	16.7 (7)
O4^iii^—Zn1—N1—C13'	−179.8 (6)	C7—C6—C8—O4	144.6 (5)
Zn1^i^—Zn1—N1—C13'	−98.8 (9)	C5—C6—C8—O3	−166.2 (5)
O3^ii^—Zn1—N1—C13	19.4 (6)	C7—C6—C8—O3	−38.3 (7)
O2^i^—Zn1—N1—C13	−69.6 (6)	C13'—N1—C9—C10	19.0 (10)
O1—Zn1—N1—C13	111.2 (6)	C13—N1—C9—C10	−0.1 (3)
O4^iii^—Zn1—N1—C13	−159.8 (6)	Zn1—N1—C9—C10	177.0 (3)
Zn1^i^—Zn1—N1—C13	−78.8 (10)	N1—C9—C10—C11	−0.2 (3)
O3^ii^—Zn1—N1—C9'	−177.8 (6)	C13'—N1—C9'—C10'	−0.1 (3)
O2^i^—Zn1—N1—C9'	93.2 (6)	Zn1—N1—C9'—C10'	177.1 (3)
O1—Zn1—N1—C9'	−86.0 (6)	N1—C9'—C10'—C11	−0.3 (3)
O4^iii^—Zn1—N1—C9'	3.0 (6)	C9—C10—C11—C12'	−18.2 (11)
Zn1^i^—Zn1—N1—C9'	84.0 (9)	C9—C10—C11—C12	0.6 (6)
O3^ii^—Zn1—N1—C9	−157.6 (6)	C9—C10—C11—C10'	82 (6)
O2^i^—Zn1—N1—C9	113.4 (6)	C9—C10—C11—C11^vi^	−176.8 (5)
O1—Zn1—N1—C9	−65.9 (6)	C9'—C10'—C11—C12'	0.7 (6)
O4^iii^—Zn1—N1—C9	23.2 (6)	C9'—C10'—C11—C12	19.4 (10)
Zn1^i^—Zn1—N1—C9	104.2 (9)	C9'—C10'—C11—C10	−87 (6)
Zn1—O1—C1—O2	13.7 (8)	C9'—C10'—C11—C11^vi^	−177.0 (6)
Zn1—O1—C1—C2	−167.4 (4)	C10—C11—C12—C13	−0.6 (8)
Zn1^i^—O2—C1—O1	−16.9 (9)	C10'—C11—C12—C13	−19.4 (12)
Zn1^i^—O2—C1—C2	164.2 (4)	C11^vi^—C11—C12—C13	176.7 (7)
O1—C1—C2—C7	152.2 (5)	C13'—N1—C13—C12	−86 (7)
O2—C1—C2—C7	−28.9 (7)	C9'—N1—C13—C12	19.2 (12)
O1—C1—C2—C3	28.7 (7)	C9—N1—C13—C12	0.1 (7)
O2—C1—C2—C3	−152.3 (5)	Zn1—N1—C13—C12	−177.0 (5)
C7—C2—C3—C4	−58.1 (6)	C11—C12—C13—N1	0.3 (9)
C1—C2—C3—C4	66.7 (6)	C10'—C11—C12'—C13'	−0.8 (8)
C2—C3—C4—C5	57.2 (6)	C11^vi^—C11—C12'—C13'	176.9 (6)
C3—C4—C5—C6	−54.6 (7)	C9'—N1—C13'—C12'	0.0 (7)
C4—C5—C6—C8	−178.4 (5)	Zn1—N1—C13'—C12'	−177.3 (5)
C4—C5—C6—C7	53.2 (6)	C11—C12'—C13'—N1	0.5 (9)
C1—C2—C7—C6	−67.6 (6)		

Symmetry codes: (i) −*x*+1/2, −*y*+3/2, −*z*+1; (ii) −*x*+1/2, *y*+1/2, −*z*+1/2; (iii) *x*, −*y*+1, *z*+1/2; (iv) −*x*+1/2, *y*−1/2, −*z*+1/2; (v) *x*, −*y*+1, *z*−1/2; (vi) −*x*+1, −*y*+2, −*z*+2.
